# MicroRNA-125b Functions as a Tumor Suppressor in Hepatocellular Carcinoma Cells

**DOI:** 10.3390/ijms13078762

**Published:** 2012-07-16

**Authors:** Hong-Yan Jia, Yu-Xuan Wang, Wen-Ting Yan, Hui-Yu Li, Yan-Zhang Tian, Shi-Ming Wang, Hao-Liang Zhao

**Affiliations:** 1 Department of Surgery, The First Hospital of Shanxi Medical University, Taiyuan 030001, China; E-Mails: swallow_jhy@163.com (H.-Y.J.); lihuiyu1978@126.com (H.-Y.L.); tyz2030@163.com (Y.-Z.T.); wangshimingSX@sina.com (S.-M.W.); 2 Department of Thoracic Surgery, Shanxi Cancer Hospital, Taiyuan 030001, China; E-Mail: wyxcardic@163.com; 3 Department of Biochemistry, Institute of Basic Medical Sciences, Chinese Academy of Medical Sciences (CAMS) & Peking Union Medical College (PUMC), National Laboratory of Medical Molecular Biology, Beijing 100005, China; E-Mail: wenting-yan@hotmail.com

**Keywords:** hepatocellular carcinoma, miR-125b, Mcl-1, IL6R

## Abstract

MicroRNAs (miRNAs) are important regulators of multiple cellular processes, and the deregulation of miRNA is a common event in diverse human diseases, particularly cancer. However, the mechanisms underlying the relationship between disordered miRNA expression and tumorigenesis have remained largely unknown. In this study, we demonstrated the down-regulation of miR-125b in hepatocellular carcinoma (HCC) tissues and HCC cell lines by Northern blot and quantitative RT-PCR analyses. The ectopic expression of miR-125b reduced the cellular proliferation and cell cycle progression of HCC cells by targeting Mcl-1 and IL6R. Furthermore, the miR-125b-induced inhibition of cell proliferation was rescued by the expression of Mcl-1 or IL6R variants that lacked 3′ UTRs. Thus, this study revealed the differential expression of miR-125b in HCC cells and elucidated its potential as a tumor suppressor in HCC development.

## 1. Introduction

MicroRNAs (miRNAs) are an abundant class of endogenous, small, non-coding regulatory RNAs, each approximately 22 nucleotides in length, which are known to regulate gene expression by binding (either perfectly or imperfectly) to the 3′ UTRs of target transcripts. Upon miRNA binding, the translation of the targeted mRNAs is repressed, and these mRNAs are rapidly degraded [1,2]. Recent studies have demonstrated that miRNAs are involved in the regulation of diverse cellular processes, including cell proliferation, apoptosis, and differentiation [3–6]. Furthermore, the deregulation of miRNAs is closely related to the pathogenesis of human cancers. For example, the epigenetic down-regulation of miR-124a contributes to the abnormal proliferation of ALL cells both *in vitro* and *in vivo* [7]. In addition, miR-31 inhibits the proliferation of serous ovarian carcinomas and other cancers [8], and the PLZF-microRNA-221/-222 pathway controls the progression of melanoma neoplasia through the down-modulation of p27Kip1/CDKN1B and the c-KIT receptor [9].

Many genome-wide miRNA profiling studies in different cancer types have highlighted the changes in miRNA expression that may be associated with cancer initiation and progression as well as specific pathologic features. Hepatocellular carcinoma (HCC) is a malignancy that has high incidence rates worldwide and is the third most common cause of cancer-related deaths [10]. Although multiple oncogenes and tumor suppressors have been identified in HCC, the role of these non-coding RNA molecules (miRNAs) in the tumor biology of HCC has only recently been addressed and remains largely unexplored. An understanding of the molecular mechanism by which miRNAs regulate HCC development may potentially provide new avenues of research that could aid in the early diagnosis and treatment of this highly malignant tumor. Recent studies have indicated the association of miRNAs in HCC, as miR-21 [11], miR-125b [12,13], miR-214 [14], miR-221 [15] *et al.* It is noteworthy that the expression of miR-125b is controversial in different types of human cancer. Therefore, the precious roles and mechanisms of miR-125b in HCC need to be further confirmed. In this study, we demonstrated that miR-125b was significantly down-regulated in human hepatocellular carcinoma patients as well as in the HCC cell lines HepG2, Huh7 and SMMC-7721. The over-expression of miR-125b in HepG2 cells inhibited cell proliferation and cell cycle progression. Furthermore, we demonstrated that Mcl-1 and IL6R were direct targets of miR-125b in HCC and that the aforementioned phenotypic changes are caused at least in part by the miR-125b-mediated targeting of Mcl-1 and IL6R. These results suggest that miR-125b functions as a tumor suppressor miRNA in HCC and is an important regulator of cellular proliferation and the cell cycle.

## 2. Results and Discussion

### 2.1. MiR-125b Expression is Down-Regulated in both HCC Tissues and HCC Cell Lines

**Figure 1:**
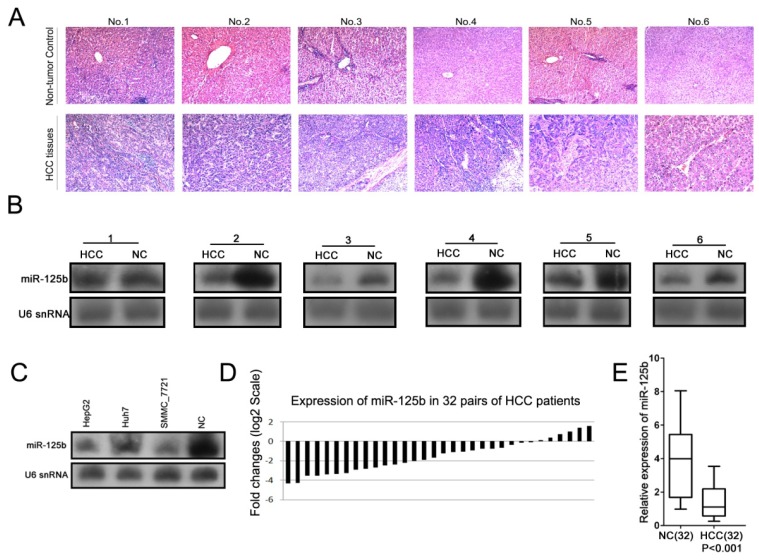
The expression of miR-125b is down-regulated in both primary hepatocellular carcinoma (HCC) tissues and HCC cell lines. (**A**) Images of HCC tissues stained with haematoxylin and eosin (H&E). (**B**) Northern blot analysis indicating a marked decrease in miR-125b in six pairs of HCC tissues (HCC) relative to matching controls (NC). (**C**) Northern blot analysis demonstrating the remarkable decrease in miR-125b expression in the HCC cell lines (HepG2, Huh7, and SMMC7721) relative to normal liver tissue (NC). (**D**, **E**) Validation of the differential expression of miR-125b in amplified HCC tissues and control cells. Panel D illustrates the expression levels of miR-125b in an independently validated set of 32 HCC patients and matched controls. The levels of miR-125b expression were quantified by real-time RT-PCR and normalized using the non-tumor control sample. Panel E provides the statistical results regarding the miR-125b expression in HCC tissues and matched controls. The P values in this panel were calculated using a nonparametric test.

A growing body of evidence indicates that miRNAs may contribute to the initiation and progression of diverse cancers by acting as either oncogenes or tumor suppressors [10,16]. The identification of cancer-related miRNAs and their targets is critical to understanding the roles of these miRNAs in tumorigenesis and may prove to be important for the development of novel therapeutic targets. To evaluate the expression of miR-125b in HCC, Northern blot analyses were performed in six pairs of poorly differentiated HCC patient tissues (Figure 1A). The expression levels of miR-125b were much lower in tumor tissues than in non-tumor tissues (Figure 1B). The examination of miR-125b expression in three HCC cell lines (HepG2, Huh, and SMMC7721) also indicated that this expression was down-regulated in tumor cell lines relative to non-tumor tissues (Figure 1C). Furthermore, quantitative real-time PCR (qRT-PCR) analysis was performed to validate the changes in the expression of miR-125b in a total of 32 pairs of matched tissue specimens (Figure 1D). The expression of miR-125b was consistently lower in HCC tissues than in matching normal liver tissues (Figure 1E). However, no significant association between miR-125b expression and clinicopathological characteristics was observed when HCC patients were divided into two groups with higher or lower expression than the controls (Supplemental Table S1). These results suggest that miR-125b may function as a tumor suppressor in HCC.

Other studies have reported controversial results regarding the expression of miR-125b in cancers. MiR-125b may act as either an oncogene or a tumor suppressor depending on the cellular context that is examined [17–23]. In prostate cancer cells, a high expression level of miR-125b stimulated androgen-independent cell growth by down-regulating Bak1 [22], whereas in breast cancer, a high expression level of miR-125b mediated the down-regulation of ERBB2 (HER2) and ERBB3 (HER3), thereby suppressing tumor growth. However, the role of miR-125b in human HCC has not been defined [23]. In our study, we identified the down-regulation of miR-125b in 32 HCC tissues as well as three HCC cell lines, suggesting a tumor suppressor role for miR-125b in HCC. The subsequent functional analysis of miR-125b in HCC cells also confirmed its tumor suppressor role in this particular cellular context.

### 2.2. MiR-125b Inhibits the Proliferation and Cell Cycle Progression of HCC Cells

**Figure 2:**
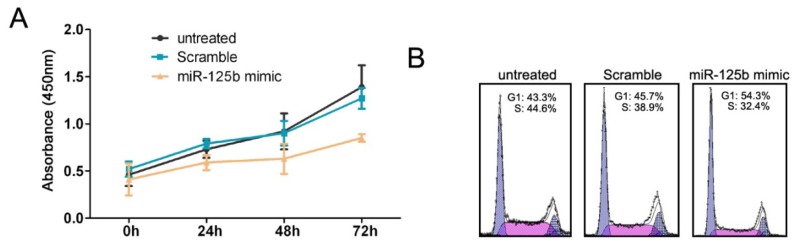
The over-expression of miR-125b inhibits the cell proliferation and cell cycle in HepG2. (**A**) The CCK-8 assay used to evaluate the proliferation of HepG2 cells after transfection with the miR-125b mimic or the scrambled oligonucleotide at different culture durations. (**B**) The cell cycle analysis of HepG2 cells treated with either the miR-125b mimic or the scrambled oligonucleotide and cultured for 24 h after cell transfection.

To understand the biological role of miR-125b in HCC, we used a miR-125b mimic to increase the intracellular levels of miR-125b in the HepG2 cell line, which has a lower level of endogenous miR-125b expression than the Huh and SMMC7721 cell lines (Figure 1C). A scrambled oligonucleotide was transfected into HepG2 as a control. The levels of miR-125b expression were much higher in the cells treated with the miR-125b mimic than in cells treated with the scrambled oligonucleotide, as revealed by the qRT-PCR analysis (data not shown). We subsequently used the CCK-8 and PI staining assays to examine the effect of miR-125b over-expression on the proliferation and cell cycle of HepG2. The enforced expression of miR-125b led to a significant decrease in the proliferation of HepG2 cells (Figure 2A). Accordingly, the percentage of S phase cells was also reduced by miR-125b over-expression (Figure 2B, Supplemental Table S2). Together, these results indicated that miR-125b could efficiently inhibit the cell proliferation and cell cycle of HepG2 *in vitro*.

### 2.3. Mcl-1 and IL6R Are Direct Targets of miR-125b in HCC

It is well known that miRNAs function by binding to specific mRNA targets, interfering with the stability of these targets and repressing their translation. As an initial step towards identifying the putative targets of miR-125b, we performed a bioinformatic screen for mRNAs with 3′ UTR sequences complementary to miR-125b. To evaluate the possible regulatory pathway of miR-125b in HCC, we used two computational algorithms, TargetScan and PicTar, to predict potential target genes of miR-125b. Of these potential targets, Mcl-1 and IL6R are two oncogenes that have been well studied in various cancers, including hepatocellular carcinoma [24–26]. Mcl-1 is an anti-apoptotic member of the Bcl-2 family that is commonly over-expressed in various types of cancers. It has been well established that the knock-down of Mcl-1 sensitizes human cancer cells to apoptosis [27,28]. IL6R is a cell surface receptor that has generally been considered to be a mediator of inflammation [29]. Recent studies have indicated that the over-expression of IL6R in human cancer cells is associated with proliferative activity, the inhibition of apoptosis, increased metastatic potential and angiogenesis [30–32].

**Figure 3:**
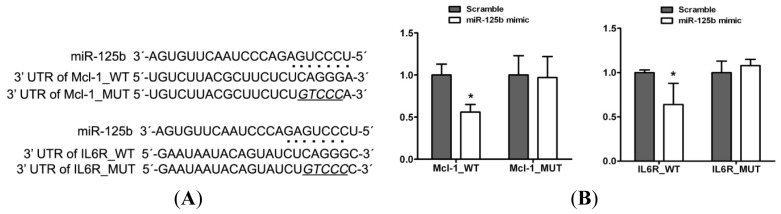

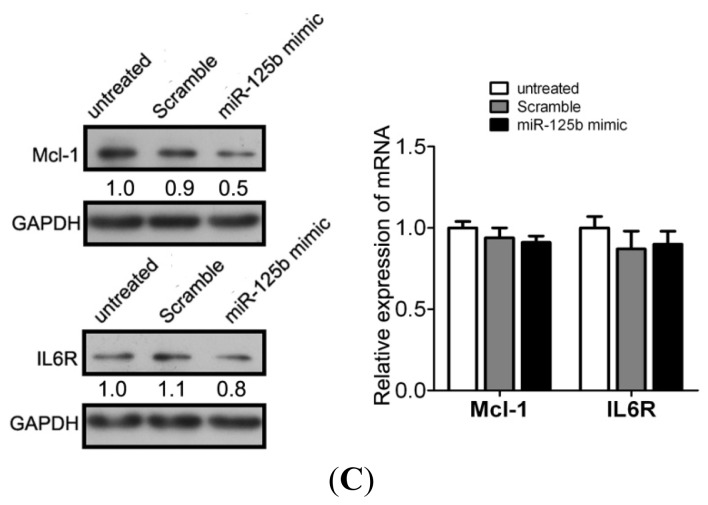
Mcl-1 and Il6R are targets of miR-125b. (**A**) The sequences of the miR-125b binding sites within the human Mcl-1 and Il6R 3′ UTRs and schematic reporter constructs. In this panel, Mcl-1_WT and IL6R_WT represent the reporter constructs containing the entire 3′ UTR sequences of Mcl-1 and IL6R. Mcl-1_MUT and IL6R_MUT represent the reporter constructs containing mutated nucleotides. (**B**) The analysis of the relative luciferase activities of Mcl-1_WT, Il6R_WT, Mcl-1_MUT, and IL6R_MUT in 293T cells. The error bars are derived from triplicate experiments, and * indicates *p* < 0.01. (**C**) Left, the immunoblotting results for Mcl-1 and Il6R in extracts from HepG2 cells transfected with either the miR-125b mimic or the scrambled oligonucleotide; right, relative expression of Mcl-1 and Il6R mRNA from HepG2 cells transfected with miR-125b mimic or scrambled oligonucleotide.

The 3′ UTR regions of Mcl-1 and IL6R were cloned into the downstream region of a luciferase reporter gene (pMIR-reporter) (Figure 3A). A reporter assay was then performed in HepG2 cells that had been co-transfected with reporter constructs and either the miR-125b mimic or the scrambled oligonucleotide. The luciferase activity was repressed in a miR-125b-dependent manner, whereas the mutation of these putative miR-125b sites abrogated the reductions in luciferase activity (Figure 3B). In accordance with the reporter assay, we observed a strong decrease in Mcl-1 and IL6R proteins in the presence of miR-125b mimics in HepG2 cells, whereas almost no effect was observed on the mRNA levels (Figure 3C). Taken together, these results provided experimental support for the concept that the expressions of Mcl-1 and IL6R were negatively regulated by miR-125b in HCC cells.

### 2.4. MiR-125b Suppresses the Expression of Mcl-1 and IL6R in HCC

**Figure 4:**
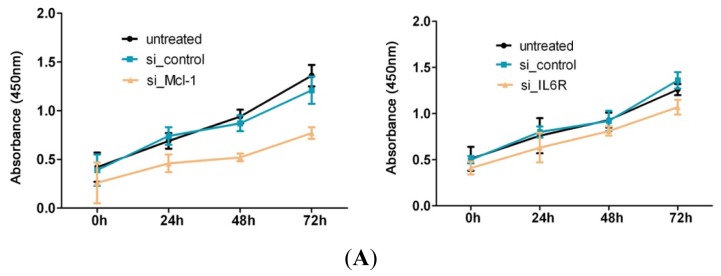

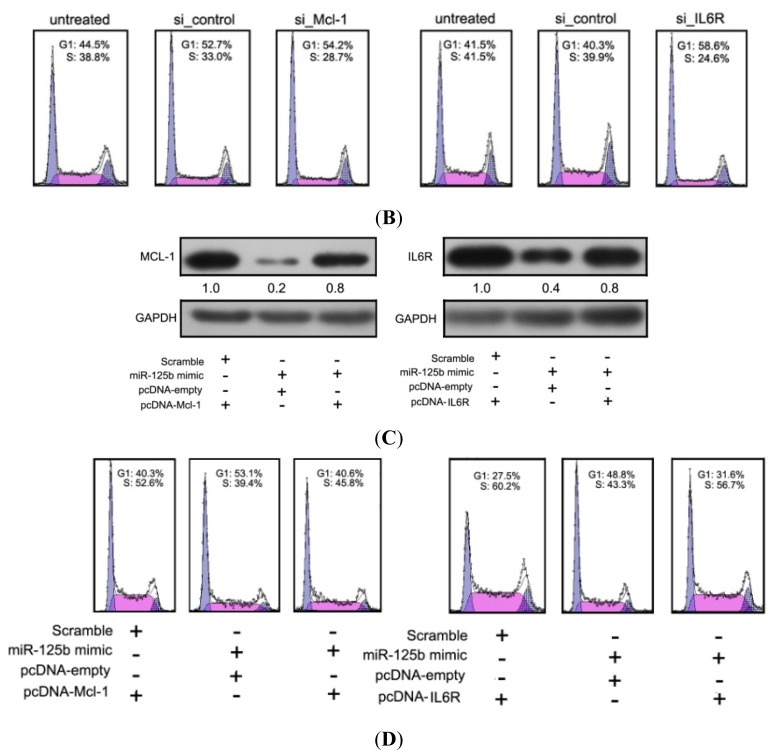
The functional relevance of miR-125b and its targets in HCCs. (**A**) Results of the CCK-8 assay for the proliferation of HepG2 cells after their transfection with either specific siRNAs targeting Mcl-1 (si_Mcl-1) and IL6R (si_IL6R) or the scrambled oligonucleotide at different culture durations. (**B**) The cell-cycle analysis of HepG2 cells treated with either specific siRNAs targeting Mcl-1 (si_Mcl-1) and IL6R (si_IL6R) or with the scrambled oligonucleotide and cultured for 24 h after cell transfection. (**C**) HepG2 cells were treated under the “rescue” condition; this panel illustrates the immunoblotting results of Mcl-1 and IL6R in extracts from HepG2 cells that were either transfected with the miR-125b mimic or the scrambled oligonucleotide for 24 h and then subsequently treated for an additional 48 h with either pcDNA-Mcl-1, pcDNA-IL6R, or pcDNA-empty. (**D**) The cell cycle analysis of HepG2 cells treated under the “rescue” condition described in the previous panel.

Because the oncogenic roles of Mcl-1 and IL6R in different cancers have been elucidated, we reasoned that the interference of Mcl-1 or IL6R expression in HCC cells might have an inhibitory effect on cell growth. As expected, the reduction in Mcl-1 and IL6R expression levels caused by the transfection of small interfering RNAs (si_Mcl-1 and si_IL6R) resulted in a marked decrease in cell proliferation and cell cycle progression, thereby indicating that the down-regulation of these two genes inhibits the proper growth of HCC cells (Figures 4A, B, Supplemental Table S3).

Although Mcl-1 and IL6R were confirmed to be targets of miR-125b and the over-expression of miR-125b in HepG2 cells altered the cell proliferation and cell-cycle progression, the direct interaction between miR-125b and target genes in the regulation of cell proliferation remains unclear. To examine whether miR-125b functions as a tumor suppressor via the direct targeting of Mcl-1 and IL6R, we adopted a “rescue” methodology to establish the functional relevance between miR-125b and its targets in HCC cells. We generated two constructs containing the full ORFs of Mcl-1 and IL6R (pcDNA-Mcl-1 and pcDNA-IL6R). The levels of both target genes (Mcl-1 and IL6R) were rescued when pcDNA-Mcl-1 and pcDNA-IL6R were transfected into HepG2 cells that had been treated with the miR-125b mimic for 24 h (Figure 4C). Consistent with the restored expression of the target proteins, increased numbers of cells at the S phase and increased cell proliferation were observed in HepG2 cells transfected with a pcDNA-Mcl-1 or pcDNA-IL6R constructs following the miR-125b mimic treatment (Figure 4D, Supplemental Table S4). Thus, we conclude that the repression of cell growth by miR-125b is typically a consequence of decreased Mcl-1 and IL6R expression in HCC.

## 3. Experimental Section

### 3.1. Human Liver Tissues and Cell Lines

This study was approved by the Committee for the Conduct of Human Research, and informed consent was obtained from all of the patients. Paired samples of HCC tissues were obtained from patients undergoing surgery and were then immediately frozen and stored in liquid nitrogen until RNA extraction. Additional information regarding the HCC subjects is provided in Supplemental Table S5. The HepG2 was obtained from the American Type Culture Collection;Huh7 and SMMC7721 were provided by the China Center for Type Culture Collection Huh7 cell line was maintained in DMEM supplemented with 10% fetal bovine serum (GIBCO, NY, USA), HepG2 and SMMC7721 were maintained in RPMI 1640 supplemented with 10% fetal bovine serum.

### 3.2. Oligonucleotides, Constructs, Cell Transfection, and Dual Luciferase Assays

MiR-125b mimic molecules (miR-125b mimic) and negative control molecules (scrambled) were obtained from Dharmacon and transfected using DharmFECT1 (Dharmacon, Austin, TX, USA) in HepG2 cells at a final concentration of 60 nM.

The Mcl-1 siRNA (si_Mcl-1), Il6R siRNA (si_IL6R), and negative control siRNA pools (si_control, D-001206-13-05) were synthesised by Dharmacon and transfected into HepG2 cells (200 nM) using DharmFECT1. The cells were cultured for 48 h and harvested for Western blot analysis as described below.

The reverse complementary sequence of miR-125b was inserted into pRL-TK downstream of the Renilla luciferase gene (Promega, WI, USA) to generate a reporter system (pRL-TK-miR-125b) to detect mature miR-125b expression in 293T cells. The 3′ UTRs of human Mcl-1 and IL6R mRNAs were amplified by PCR and cloned into pRL-TK to generate the Mcl-1 reporter (Mcl-1_WT) and IL6R reporter (IL6R_WT). The sequences of the primers that were used for 3′ UTR amplification are provided in Supplemental Table S6. Mutations in these two mRNA sequences were created using the QuikChange Site-Directed Mutagenesis kit (Stratagene, CA, USA). The 293T cells were co-transfected with 0.4 μg of the reporter construct, 0.2 μg of pGL-3 control vector, and miR-125b mimic or scrambled controls. The cells were harvested at 24 h post-transfection and assayed using the Dual Luciferase Assay (Promega, WI, USA) according to the manufacturer’s instructions. All of the transfection assays were conducted in triplicate.

### 3.3. The Rescue Assays of Mcl-1 and IL6R Gene Expression

The full-length Mcl-1 and IL6R ORFs were amplified by PCR and cloned into pcDNA3.1 to generate the pcDNA-Mcl-1 and pcDNA-IL6R constructs that were used in the rescue assays. The sequences of the primers used for the full-length amplifications are provided in Supplemental Table S6. HepG2 cells in six-well plates were first transfected with the miR-125b mimic or the scrambled oligonucleotide (60 nM). After 24 h in culture, these HepG2 cells were then co-transfected with miR-125b mimic (30 nM) and 2.0 μg of either pcDNA-Mcl-1, pcDNA-IL6R, or pcDNA-empty. The cells were harvested at predetermined intervals and assayed as necessary.

### 3.4. RNA Isolation, Northern Blots and Western Blots

Total RNA was extracted from cells and tissues using TRIzol (Invitrogen). The Northern blot analysis of miRNAs was performed in accordance with previously published methods [16]. Western blot analysis was also performed in accordance with previously published methods [33], and the membranes were blotted with antibodies for Mcl-1, IL6R and GAPDH that were obtained from Santa Cruz (CA, USA).

### 3.5. Quantitative Real Time RT-PCR

The RNA was quantified by assessing its absorbance at 260 nm. The cDNA was synthesized from 2 μg of total RNA using M-MLV reverse transcriptase (Invitrogen). As described by Chen, stem-loop RT primers were used for the reverse transcription of miRNAs. Quantitative RT-PCR was conducted using the ABI PRISM 7500 real-time PCR System (Applied Biosystems, CA, USA). For the PCR amplification, the SYBR Premix Ex Taq kit (Takara, Dalian, China) was utilized according to the manufacturer’s instructions. For miRNAs, U6 snRNA was used as the endogenous control. The sequences of the primers used for the reverse transcription and quantitative PCR are provided in Supplemental Table S6. The comparative Ct method was used to quantify target genes relative to their endogenous control. For each individual analysis, one of the samples was designated as the calibrator and given a relative value of 1.0; all of the quantities were then expressed as n-fold relative to the calibrator.

### 3.6. Cell Proliferation Assay

The proliferation of the transfected HepG2 cells was measured with a Cell Counting Kit-8 (Dojin Laboratories, Kumamoto, Japan). Briefly, cells were seeded in 96-well plates (200 μL/well) at a density of 1 × 10^4^ cells/well. CCK-8 (20 μL) was added to the wells at 0, 24, 48, and 72 h post-transfection, and the plates were incubated at 37 °C for an additional 4 h. The absorbance values were determined at 450 nm using an automated ELISA plate reader. The average percentages of cells in G1 and S phase are shown in Supplemental information, Table S2 (corresponding to Figure 2B), Table S3 (corresponding to Figure 4B), Table S4 (corresponding to Figure 4D).

### 3.7. Flow Cytometry

For the detection of intracellular DNA, harvested cells were washed twice with PBS and fixed in 75% ethanol at 4 °C overnight. Following two washes with ice-cold PBS, the fixed cells were first incubated in RNase A (20 μg/mL) at 37 °C for 30 m and then stained with propidium iodide (PI, 0.5 mg/mL) at 4 °C for 30 m. Subsequently, the cells were washed with BSA/PBS (1%) and resuspended in 500 μL of PBS. Flow cytometry data for PI detection were acquired from approximately 10^5^ cells using a Becton-Dickinson flow cytometer. All of the assays were conducted in triplicate.

### 3.8. Statistical Analysis

A two-tailed Student’s *t*-test was performed to analyze the data. *p*-values < 0.05 were considered to be significant.

## 4. Conclusions

In this study, we evaluated the expression of miR-125b in HCC tissues and cell lines and explored the biological role and regulatory mechanisms of miR-125b in tumorigenesis. We demonstrated that miR-125b was down-regulated in HCC tissues and cell lines and was able to inhibit the cell proliferation and cell cycle progression of HCC cells by targeting Mcl-1 and IL6R. Our results are consistent with Liang’s study, which indicated that miR-125b exerts tumor-suppressive effects in hepatic carcinogenesis through the suppression of oncogene LIN28B expression [13]. Moreover, we revealed a novel regulatory pathway linking miR-125b to Mcl-1 and IL6R in HCC. Mcl-1 is the anti-apoptotic protein, which regulates intrinsic apoptosis induction at the mitochondrial level, and is often over-expressed in human cancers. The up-regulation of Mcl-1 in HCC cells could inhibit chemotherapeutic drug-induced apoptosis of tumor cells [34]. The IL6R-mediated signal pathway regulates cell growth and differentiation and plays an important role in immune response. Chronic hepatitis B or C viral infections are major risk factors of HCC, and up-regulation of IL6R could significantly differentiate HCC from hepatitis patients [35]. Our findings identify miR-125b as a potent regulator of Mcl-1 and IL6R, which may provide a novel therapeutic strategy for treatment of HCC and other Mcl-1 or Il6R-driven cancers. These results confirme the tumor suppressive role of miR-125b in HCC and provide evidence for the potential usefulness of miR-125b in miRNA-based cancer therapy.

## Supplementary Information
