# Supplementary material for: MicroRNA-125b Functions as a Tumor Suppressor in Hepatocellular Carcinoma Cells
Source: Int J Mol Sci. 2012 Jul 16;13(7):8762–74. doi: 10.3390/ijms13078762 (PMC3430264; doi:10.3390/ijms13078762)
Supplement: Supplementary file 1 [file ijms-13-08762-s001.pdf]

MicroRNA-125b Functions as a Tumor Suppressor in Hepatocellular Carcinoma Cells

Supplemental Information

Table S1. Relationship between clinicopathological factors and low expression of miR-125b in HCC tissues.

Variables	Patients, *n*		*p* value
	Low miR-125b (*n* = 26)	High miR-125b (*n* = 6)	
**Gender**			0.62
male	20	4	
female	6	2	
**Age**			0.67
<57	10	3	
≥57	16	3	
**AFP (ng/mL)**			1
<100	11	2	
≥100	15	4	
**Grading**			0.126
G1	5	3	
G2	5	2	
G3	16	1	

Table S2. Average values from the cell cycle analysis of HepG2 cells transfected with miR-125b mimic or Scramble.

Samples	**Percentage of cells in different phases of cell cycle**
untreated	G1: 45.7 ± 2.2%<br>S: 43.7 ± 0.8%
Scramble	G1: 45.3 ± 3.0%<br>S: 37.8 ± 2.0%
miR-125b mimic	G1: 54.8 ± 1.2%<br>S: 31.5 ± 1.2%

Table S3. Average values from the cell cycle analysis of HepG2 cells transfected with siRNAs or negative control.

Samples	**Percentage of cells in different phases of cell cycle**
untreated	G1: 42.5 ± 1.8%<br>S: 38.8 ± 1.4%
si_control	G1: 49.2 ± 3.1%<br>S: 35.5 ± 2.2%
si_Mcl-1	G1: 56.9 ± 2.3%<br>S: 27.7 ± 0.9%
untreated	G1: 42.2 ± 3.9%<br>S: 42.0 ± 2.4%
si_control	G1: 41.6 ± 1.8%<br>S: 40.5 ± 1.1%
si_IL6R	G1: 56.1 ± 2.3%<br>S: 25.3 ± 1.2%

Table S4. Average values from the cell cycle analysis of HepG2 cells in miR-125b rescue assay.

Samples	**Percentage of cells in different phases of cell cycle**
Scramble + pcDNA-Mcl-1	G1: 40.3 ± 1.6%<br>S: 51.9 ± 2.1%
miR-125b mimic + pcDNA-empty	G1: 55.6 ± 2.4%<br>S: 39.0 ± 2.1%
miR-125b mimic + pcDNA-Mcl-1	G1: 42.8 ± 1.9%<br>S: 47.5 ± 2.1%
Scramble + pcDNA-Mcl-1	G1: 29.8 ± 2.0%<br>S: 56.9 ± 2.9%
miR-125b mimic + pcDNA-empty	G1: 49.2 ± 1.3%<br>S: 43.3 ± 1.4%
miR-125b mimic+ pcDNA-Mcl-1	G1: 31.9 ± 1.4%<br>S: 57.4 ± 1.7%

Table S5. Description of 32 HCC cases.

Sample ID	Gender	Age	AFP (ng/mL)	Grading
1	Male	51	4412.65	G3
2	Male	55	84.51	G3
3	Male	39	387.54	G3
4	Male	42	694.11	G3
5	Female	47	1105.21	G3
6	Male	58	16.47	G3
7	Male	62	426.58	G1
8	Male	59	55.43	G1
9	Female	52	6.97	G3
10	Male	55	871.34	G2
11	Male	46	4349.55	G2
12	Female	57	644.12	G3
13	Male	54	16.54	G3
14	Male	64	6.98	G2
15	Male	63	7.39	G1
16	Male	60	846.97	G1
17	Female	59	8.46	G1
18	Female	47	8.43	G2
19	Male	58	1156.49	G3
20	Male	64	8789.64	G3
21	Male	68	10023.37	G3
22	Male	64	184.56	G2
23	Male	64	546.25	G3
24	Female	59	1698.44	G3
25	Male	62	12.10	G3
26	Male	67	8.49	G3
27	Male	56	75.64	G1
28	Male	57	4641.23	G1
29	Female	52	144.29	G2
30	Female	46	165.47	G1
31	Male	66	138.62	G2
32	Male	67	83.05	G3

Table S6. Primers used for DNA cloning and qRT-PCR, and probes used for Northern blot.

**Primers for DNA cloning**	**Sequence (5'–3')**
miR-125b reporter up	CTAGATCACAAGTTAGGGTCTCAGGGAA
miR-125b reporter down	CGCGTTCCCTGAGACCCTAACTTGTGAT
Mcl-1_WT up	TCTAGACCCAATTCATTAGGTATGACTG
Mcl-1_WT down	ACGCGTGTTAGGGAAACACACTACATTTG
Mcl-1_MUT up	CTGTCCCAAAAACATGCAGTCCTCTAG
Mcl-1_MUT down	TTTTGGGACAGAGAAGCGTAAGAC
IL6R_WT up	TCTAGAGCTAGAGTGAACTTGGGCCAC
IL6R_WT down	ACGCGTCACAGCGAATATTGGATATTCCACC
IL6R_MUT up	GTCCCCCTGGTCGTTTTCAACAGA AT
IL6R_MUT down	CAGGGGGACAGATACTGTATTATTC
**Primers for qRT-PCR**	
miR-125b RT	GTCGTATCCAGTGCAGGGTCCGAGGTATTCGCAC TGGATACGACTCACAAG
miR-125b up	GCGCTCCCTGAGACCCTAA
miR-125b down	CAGTGCAGGGTCCGAGGT
U6 snRNA RT	AAAATATGGAACGCTTCACGAATTTG
U6 snRNA up	CTCGCTTCGGCAGCACATATACT
U6 snRNA down	ACGCTTCACGAATTTGCGTGTC
**Probes for Northern blot**	
miR-125b	TCACAAGTTAGGGTCTCAGGGA
U6 snRNA	CACGAATTTGCGTGTCATCCTTGC

All data were obtained from three independent experiments.
